# Systematic examination of methodological inconsistency in operationalizing cognitive reserve and its impact on identifying predictors of late-life cognition

**DOI:** 10.1186/s12877-023-04263-9

**Published:** 2023-09-09

**Authors:** Kerry A. Howard, Lauren Massimo, Sarah F. Griffin, Ryan J. Gagnon, Lu Zhang, Lior Rennert

**Affiliations:** 1 Department of Public Health Sciences, Clemson University, Clemson, SC 29634 USA; 2 Center for Public Health Modeling and Response, Clemson University, Clemson, SC 29634 USA; 3 Department of Neurology, Penn Frontotemporal Degeneration Center, University of Pennsylvania, Philadelphia, PA USA; 4 School of Nursing, University of Pennsylvania, Philadelphia, PA USA; 5 Department of Parks, Recreation and Tourism Management, Clemson University, Clemson, SC USA

**Keywords:** Cognitive Reserve, Aging, Cognitive Aging, Methodology, Operationalization

## Abstract

**Background:**

Cognitive reserve (CR) is the ability to maintain cognitive performance despite brain pathology. CR is built through lifecourse experiences (e.g., education) and is a key construct in promoting healthy aging. However, the operationalization of CR and its estimated association with late-life cognition varies. The purpose of this study was to systematically examine the operationalization of CR and the relationship between its operationalization and late-life cognition.

**Methods:**

We performed a comprehensive review of experiences (proxies) used to operationalize CR. The review informed quantitative analyses using data from 1366 participants of the Memory and Aging Project to examine 1) relationships between proxies and 2) the relationship between operationalization and late-life cognition. We also conducted a factor analysis with all identified CR experiences to create a composite lifecourse CR score. Generalized linear mixed models examined the relationship between operationalizations and global cognition, with secondary outcomes of five domains of cognition to examine consistency.

**Results:**

Based on a review of 753 articles, we found the majority (92.3%) of the 28 commonly used proxies have weak to no correlation between one another. There was substantial variability in the association between operationalizations and late-life global cognition (median effect size: 0.99, *IQR*: 0.34 to 1.39). There was not strong consistency in the association between CR operationalizations and the five cognitive domains (mean consistency: 56.1%). The average estimate for the 28 operationalizations was 0.91 (*SE* = 0.48), compared to 2.48 (*SE* = 0.40) for the lifecourse score and it was associated with all five domains of cognition.

**Conclusions:**

Inconsistent methodology is theorized as a major limitation of CR research and barrier to identification of impactful experiences for healthy cognitive aging. Based on the weak associations, it is not surprising that the relationship between CR and late-life cognition is dependent on the experience used to operationalize CR. Scores using multiple experiences across the lifecourse may help overcome such limitations. Adherence to a lifecourse approach and collaborative movement towards a consensus operationalization of CR are imperative shifts in the study of CR that can better inform research on risk factors related to cognitive decline and ultimately aid in the promotion of healthy aging.

**Supplementary Information:**

The online version contains supplementary material available at 10.1186/s12877-023-04263-9.

## Introduction

Cognitive decline that occurs with aging includes changes in multiple domains, such as memory and executive function, and can contribute to functional deficits [1]. As the population lives longer and large generations enter their later adult years, cognitive deficits are expected to intensify their burden on families and the healthcare system, creating a major public health concern [2–4]. The rate of cognitive decline is highly variable from individual to individual [5, 6]. Although there are likely multiple factors that contribute to the variability of cognitive decline in older adults, cognitive reserve (CR) has been put forth as an important contributor to successful cognitive aging. The CR hypothesis theorizes that individuals encounter experiences throughout the lifecourse that may produce varying levels of protective benefits, increasing the brain’s adaptability to age-related brain changes and allowing for maintainance of higher cognitive function [7, 8]. Therefore, individuals with high CR have delayed onset of symptoms of cognitive decline, despite the presence of brain pathology. However, collectively, approaches to the operationalization of CR through proxies, such as education and occupational attainment, vary between studies, perhaps creating inconsistencies in the relationship between CR and late-life cognition [9, 10]. As the field of aging moves forward, a systematic examination of CR operationalization and a better understanding of the relationship between CR operationalization and late-life cognition is necessary to facilitate research to discern how to slow cognitive decline through CR promoting activities.

CR cannot be directly measured due to its theoretical nature and, therefore, the study of CR is open to threats to construct validity [10, 11]. As a result, studies examining CR must operationalize it, or define it in a way that can be measured. The most common operationalization of CR relies on proxy measures. Proxies are experiences that are hypothesized to contribute to the development of CR [12]. Common proxies include education, occupation, and cognitively-stimulating activities, among a large variety of considered experiences. However, the association between these proxies and their relative importance to late-life cognition remain unclear. For example, while eduction is frequently used as a measure of CR, a systematic review found a lack of consensus for its protective effect [13], and others have debated the utility of educational attainment relative to other experiences, such as occupational attainment [14, 15]. A portion of variance may stem from measurement differences. Education is most often measured as years of formal education, while some argue that quality may be more indicative of CR, and perhaps more important, than years [9, 16, 17]. Occupation, another widely-used CR proxy, has been measured based on level (e.g., professional worker, service worker) [18, 19], or based on the occupation’s cognitive demands through the Occupational Information Network (O*NET) [20, 21]. The variety of proxies may be problematic through a resulting failure to produce a consistent representation of important life experiences.

Furthermore, the timing of proxies within a person’s life produce additional measurement challenges and alternatives. Many studies investigating the relationship between CR and late-life cognition rely on experiences from single life timepoints (e.g., late-life physical activity); however, at its foundation, the CR hypothesis attributes the development of higher CR to experiences across the lifecourse [12, 22]. In a set of studies, Xu and colleagues developed lifespan CR scores, an accummulation of multiple variables: education; early-, mid-, and late-life cognitive activities; and late-life social activities [23, 24]. Their results showed that a higher lifespan CR score reduced risk of cognitive outcomes. Similarly, a number of questionnaires have been developed to produce cumulative CR scores, including the Cognitive Reserve Index questionnaire (CRIq) [25] and the Lifetime of Experience Questionnaire (LEQ) [26]. The CRIq focuses on total years of education, years of different types of labor, and years of activities [25]; and the LEQ asks participants to recall participation in activities at three different life timepoints, young adult (13–30), adult (30–65), and older adult (from 65 on) [26]. As such, the approaches used to examine the relationship between CR and late-life cognition show wide variability.

The purpose of the present study was to explore characteristics of CR proxies commonly used in the literature, investigate the relationship between different proxies, evaluate the impact of CR operationalization on its association with late-life cognition, and examine operationalizations of CR which incorporate multiple lifecourse experiences. We extracted proxies that have been used in the published literature to operationalize CR through a comprehensive review of the literature. Findings from the review were utilized to inform quantitative analysis using data from the Rush Memory and Aging Project (MAP) to investigate the operationalization of CR and how operationalization relates to understanding late-life cognition.

## Methods

### Comprehensive literature search

We assessed articles published between January 1990 and December 2021 [27]. All searches were done in January of 2022 through three search engines, PsycInfo, Web of Science, and PubMed, and excluded special populations and study designs other than cohort and cross-sectional [27, 28]. The search was undertaken in two phases: an initial search for possible proxies and a follow-up search of each proxy to determine prevalence of that proxy among the CR literature and qualities of its use. Additional details of the literature search, including inclusion criteria and PRISMA flowchart [29], are provided in Appendix 1. After assessment of 5326 screened articles, 1412 of which required retrieval, 753 articles met inclusion criteria. Experiences used as proxies (e.g., reading, going to the library) were grouped into 20 proxy categories based on their use in the literature. For example, researchers may examine playing games, reading, and going to the library all under the category of cognitive activities. These categories were agreed upon by two researchers. Total number of articles retrieved and final inclusion number by proxy categories is shown in Table 1.

**Table 1:** Literature search frequency results by each proxy category

Proxy Categories	Number Retrieved	Number Meeting Inclusion Criteria
Education	501	280
Occupation	155	91
Leisure activities	76	56
Cognitive activities	131	45
Mood and personality	84	42
Intelligence quotient	72	39
Bilingualism	43	29
Physical activities	59	29
Socioeconomic status	70	29
Physical function	42	24
Social activities	45	21
Nutrition	22	15
Marriage status	17	11
Social network	14	11
Substances	20	9
Parental education	14	8
Social support	13	6
Retirement	27	5
Novelty	4	2
Adverse childhood experiences	3	1
**Total**	**1412**	**753**

CR proxy use in the literature varied widely. Education was the most common proxy category by a large margin (*N* = 280), with over three times that of the second-most common, occupation (*N* = 91). Education and occupation were used together most frequently of any pairing (*N* = 58). Leisure activities (*N* = 29), cognitive activities (*N* = 26), and premorbid intelligence quotient (IQ) (*N* = 24) were also frequently used with education. Additionally, several proxies were measured in different ways. Occupation was split almost equally in being categorized based on one’s level (54.76%) or based on qualities of the work (45.24%). On the other hand, IQ was divided among a larger variety of measures, with the National Adult Reading Test (NART) at 46.15%; verbal or vocabulary ability, including the Wide Range Achievement Test (WRAT), at 28.21%; literacy level at 20.51%; and formal IQ at 5.13%. Leisure activities and social activities often showed overlapping components or experiences.

Information from the comprehensive literature review about prevalence and qualities of proxies were used to inform quantitative analysis exploring (1) the relationships between proxies through a correlation and multiphase confirmatory factor analysis and (2) the relationship between differing operationalization and late-life cognition.

### Data source and participants

Data was obtained from the Rush Memory and Aging Project (MAP). MAP is an ongoing, hybrid cohort study that recruits participants ≥ 65 years of age, without known dementia, from retirement communities in Illinois, United States [30]. At baseline, participants were asked about history of activities throughout their lives. Participants underwent yearly follow-up, including tests for cognitive functioning. The MAP dataset included 2131 individuals. Individuals presenting with mild cognitive impairment (MCI) or Alzheimer’s disease (AD) at baseline were removed from the analysis, resulting in a final sample of 1459 healthy participants. MAP has been approved by the Institutional Review Board of Rush University Medical Center.

### CR operationalization techniques

#### Proxies

The MAP dataset contained variables related to 18 of the 20 proxy categories from the literature search, with no reasonable equivalents for nutrition and novelty. Observed variables in MAP (life experiences) were used to create composite scores relating to the proxy category (see Table 2). Composite scores were based on frequency of activities for leisure activities, cognitive activities, and social activities. Late-life leisure activities and all cognitive and social activities were rated on a frequency scale from 1 (once a year or less) to 5 (every day or about every day). The three variables which retrospectively comprised leisure activities at age 18 (attending concerts, plays, or musicals; taking music lessons; and taking art, dance, or theatre lessons) were coded as 0 (have not attended any or taken any lessons) and 1 (have attended or taken lessons). Social network and physical function were defined by multiple variables, while different ages provided multiple measurements for leisure activities, cognitive activities, and socioeconomic status. Primary life occupation was classified based on U.S. census categories and based on cognitive demands through O*NET, a database of job characteristics sponsored by the United States Department of Labor. As described elsewhere [18, 19], census classification used a rank from 1–6: 1 = no occupation; 2 = farm workers; 3 = unskilled laborers; 4 = operative and service workers; 5 = craftsmen, managers, administrators, and sales; and 6 = professional and technical workers. Using established scores from O*NET, we also created an occupational cognitive requirements score (OCRS) for each participant [21]. Details for the calculation of the OCRS are described elsewhere [21] and in Appendix 2. The 18 available proxy categories, in addition to multiple measurements for social network, physical function, leisure activities, cognitive activities, socioeconomic status, and occupation resulted in 28 different forms of operationalization using proxy categories, including two levels for job attainment (professional and managerial compared to operative and service workers). All variables were coded such that higher values indicated a better score or better health (e.g., more mobility, less symptoms of depression).

**Table 2:** Cognitive reserve operationalization and available MAP variables used in analyses

**Proxy Categories for Cognitive Reserve Operationalization**	**Corresponding Observed Variables form MAP**
Education	Years of formal education
Occupation	
Attainment level	Job attainment level (professional, managerial, operative and service workers)
Cognitive requirements	Occupational cognitive requirements score
Leisure activities	Frequency of activities
Age 18	Attending concerts, plays, or musicals; taking music lessons; taking art, dance, or theatre lessons
Late life	Volunteering, traveling
Cognitive activities	Frequency of activities
Age 6	Playing games, reading, hearing stories
Age 12	Playing games, reading, going to the library, reading the newspaper, reading magazines, writing letters, playing team sports
Age 18	Playing games, reading, going to the library, reading the newspaper, reading magazines, writing letters
Age 40	Playing games, reading, going to the library, reading the newspaper, reading magazines, writing letters
Late life	Playing games, reading, going to the library, reading the newspaper, reading magazines, writing letters
Mood and personality	Depression symptoms [31]
Intelligence quotient	National Adult Reading Test [32]
Bilingualism	Years of training in a foreign language
Physical activities	Frequency of physical activity in late life
Socioeconomic status	
Age 40	Income level
Late life	Income level
Physical function	
Mobility	Mobility [33]
Body measurements	Body mass index
Social activities	Frequency of activities in late life: participation in clubs and groups, visiting relatives, going to church, going to restaurants or sporting events
Marriage status	Marriage status in late life
Social network	
Size	Social network size
Isolation	Perceived social isolation in late life
Substances	Any history of smoking
Parental education	Average years of formal education for father and mother
Social support	Perceived social support
Retirement	Retirement status in late life
Adverse childhood experiences	Total adverse experiences during childhood

#### Questionnaires

We created composite scores based on the CRIq and the LEQ. While the two questionnaires could not be mimicked exactly due to incompatibility of some MAP variables with the questionnaires’ items (e.g., years of education rather than different training), we were able to base the composite score on the types of experiences that are included in each questionnaire. The CRIq-based score was a combination of education, job attainment level, children, frequency of participation in leisure activities at age 18, reading magazines and newspapers at the age with the highest frequency, and the following in late life: seeing relatives, participation in groups, going to church, going to restaurants and sporting events, traveling, and volunteering. The LEQ-based score was divided into three ages: ages 13–30 employed the variables of education, taking music lessons, and reading; ages 30–65 employed the variables of job attainment level and reading; and age 65 and older employed the variables of retirement, participation in groups and clubs, volunteering, going to restaurants and sporting events, visiting relatives, traveling, physical activities, and reading. Reading included books, newspapers, and magazines. Weights were applied to the variables based on the scoring guide for the CRI-q [25] and a validation of the LEQ in an American population [34].

#### Development of Lifecourse CR score

A lifecourse CR score was assigned to each participant using variables from MAP. The score was developed through a multiphase confirmatory factor analysis (CFA). Observed variables were grouped and evaluated under six overarching dimensions based on prior uses of MAP variables: Leisure Activities, Social Characteristics/Activities, Physical Characteristics/Activities, Cognitive Activities, Socioeconomic Status (SES), and Demographics/Personality [23, 30, 35, 36]. Four variables were examined under more than one dimension to assess best fit: volunteering in late life, going to restaurants and sporting events in late life, foreign language training, and team sports participation at age 12. Additional detail is provided in the Statistical Analyses Subsection.

A list of dimensions, factors within each dimension, and observed variables loaded to those factors is provided in Table 3. Physical Characteristics/Activities (late-life physical activities, mobility, and BMI) and Demographics/Personality (marriage status, retirement status, number of depression symptoms, and smoking status in late life) were underspecified and thus excluded from the factor analysis.

**Table 3:** Dimensions, factors, and observed variables of preliminary confirmatory factor analysis models

Dimensions	Factors	Observed Variables
Leisure Activities	Age 18 experiences	Attending concerts, plays, or musicals
		Taking music lessons
		Taking art, dance, or theatre lessons
	Late life experiences	Volunteering
		Traveling
		Going to restaurants or sporting events
Social Characteristics/Activities	Late life experiences	Participation in clubs and groups
		Visiting relatives
		Going to church
		Going to restaurants or sporting events
		Volunteering
	Social features	Social network size
		Perceived social isolation
		Perceived social support
		Playing team sports at age 12
Cognitive Activities	Age 6 experiences	Playing games
		Reading
		Hearing stories
	Age 12 experiences	Playing games
		Reading
		Going to the library
		Reading the newspaper
		Reading magazines
		Writing letters
		Playing team sports
	Age 18 experiences	Playing games
		Reading
		Going to the library
		Reading the newspaper
		Reading magazines
		Writing letters
		Years of foreign language training through age 18
	Age 40 experiences	Playing games
		Reading
		Going to the library
		Reading the newspaper
		Reading magazines
		Writing letters
	Late life experiences	Playing games
		Reading
		Going to the library
		Reading the newspaper
		Reading magazines
		Writing letters
Socioeconomic Status	Parental, genetic, youth	Adverse childhood experiences
		Intelligence quotient
		Parental education
		Years of foreign language training through age 18
		Playing team sports at age 12
	Adult experiences	Job attainment level
		Education
		Income at age 40
		Income in late life

### Cognition

Cognitive scores for each participant were used as outcomes in analyses examining the association between operationalization of CR and late-life cognition. As previously described [37, 38], participants completed a battery of 19 cognitive tests annually which examined five cognitive domains: episodic memory, perceptual orientation, perceptual speed, semantic memory, and working memory. Details on tests and scoring are given in Appendix 3. In addition to the domains, all test scores are averaged to create a composite score for global cognition [35, 39, 40]. The primary outcome in the present analyses was global cognition across follow-up. We also used each of the five domains as secondary outcomes, in order to assess the consistency of the CR operationalization techniques. While NART has been combined into cognitive score composites and the semantic memory domain in other uses of MAP data with these outcomes, we excluded NART from the domain and global scores in order to use it as a predictor as the IQ CR proxy [35], given its use as an estimate for premorbid IQ [41].

### Statistical analyses

#### Correlation

We examined the correlation 1) between CR proxy categories, 2) between observed variables or experiences in the same proxy category when different variables for measurement were used within the same category (leisure activities, cognitive activities, and social activities), and 3) between observed variables that overlapped categories in the literature (variables included in leisure activities and social activities). Due to the categorical natural of variables, Spearman’s rank order correlations were applied for these analyses. Data analysis was completed using SAS software version 9.4. Strength of the correlation was based on the value of Spearman’s rho (*ρ*) and boundaries defined by Cohen (1988) [42] with small, moderate, and large defined as *ρ* ≥ 0.1, *ρ* ≥ 0.3, and *ρ* ≥ 0.5, respectively. 

#### Multiphase confirmatory factor analysis

Multiphase confirmatory factor analysis was used to compute the lifecourse CR score. Each dimension went through multiple iterations in which factor loadings (λ) for each variable were assessed for the strength of the relation between the variable and latent factor [43, 44]. A single variable was removed from the factors after each iteration until all variables had a factor loading (λ) ≥ 0.4. A value of 0.4 was chosen as < 0.4 is considered low item loading, indicative of poor fit [43, 45]. Additionally, a cut-off near 0.5 was preferred because 0.5 indicates that the variable is reflecting more variance than within-factor error and a value of 0.4 was ultimately chosen in favor of variable conservation. A robust maximum likelihood technique was utilized throughout because it is superior to simple maximum likelihood at producing reliable estimates despite potential data uncertainty, such as missing data and nonnormality due to data not measured on a continuous scale (e.g., frequency of activities) [46, 47]. Collectively, model fit was examined through a combination of model chi-square (χ^2^), the robust version of the Comparative Fit Indices (CFI), the robust version of the Root Mean Squared Error of Approximation (RMSEA), and the Standardized Root Mean Square Residual (SRMR). Levels closer to one for CFI (i.e., ≥ 0.95); and levels closer to zero for RMSEA (i.e., ≤ 0.07) and SRMR (i.e., < 0.08) were indicative of better model fit [44, 45, 48–51]. Chi-square, CFI, RMSEA, and SRMR are the recommended indices to be reported [44]. In cases in which all variables showed a loading ≥ 0.4, but the model fit was weak (i.e., failing to reflect sufficient factor loading levels as theorized), we examined model indices to find errors using a combination of empirical and theoretical justification for covarying errors.

Once the above criteria were met, the dimensions were combined into a single overall measurement model, which similarly went through multiple iterations to retain loadings ≥ 0.4. Finally, a predicted value of the latent, overall factor of CR was obtained as a weighted composite of the factors retained in the final CFA model, such that a unique lifecourse CR score could be assigned to each participant [23]. All analyses were conducted with R software version 4.2.1, implementing the Lavaan package (version 0.6–12) [52].

#### Generalized linear mixed effects models

We used generalized linear mixed effects models to investigate the association between each of the CR operationalizations techniques and late-life cognition. In total, 28 CR operationalizations based on proxy categories, two operationalizations based on questionnaires, and one lifecourse CR score from the factor analysis resulted in 31 CR operationalization techniques for analysis. We examined the relationship between each CR operationalization technique and cognitive scores over the course of follow-up. Continuous predictor variables were standardized to a mean of 0 and a standard deviation of 1 for straightforward comparison of effect sizes. One CR proxy, job attainment level, was ordinal with three levels: operative and service workers (reference group), managerial jobs, and professional jobs. Each CR proxy, questionnaires score, and lifecourse CR score were modeled separately. All models included (linear) time and adjusted for age at study entry, sex, race, ApoE4 genotype (a genetic risk factor for AD), and baseline Mini-Mental State Exam score. Models incorporated a random slope for time in follow-up years and a random intercept, while accounting for repeated measures on the same participant. Analyses were performed using SAS software version 9.4. A Bonferroni correction for multiple comparisons was applied to evaluate the statistical significance, such that *p* < 0.0016 was considered statistically significant, thus reducing potential Type 1 error rates.

## Results

### Baseline characteristics of study population

Of 1459 MAP participants who presented as healthy at baseline (had not been diagnosed with MCI or AD), 93 participants were excluded because they did not have or provide any follow-up data. Therefore, the sample for the present study’s analyses consisted of 1366 participants. Characteristics of the sample are in Table 4. The average age at entry was 79.5 (*SD* = 6.51) years. Most identified as women (76.50%) and Non-Hispanic White (91.29%). On average, participants had 15 years of education. Most had done managerial-level work (53.19%) and a high percent (88.36%) had retired from their job by study entry. Few participants had copies of ApoE4 (20.68%). There was a small amount of missing data across all variables (7.10%). While there is not a consensus on an acceptable amount of missing data, < 10% is unlikely to bias the results if there is not a pattern to the missingness [53–55]. Based on examination of the relationship between observed experience variables in the dataset and dummy variables indicative of missing or nonmissing, there was not evidence of a pattern to the missingness [55].

**Table 4:** Characteristics of study population

**Variable**	***M(SD) or N(%)***
Age at study entry, *M(SD)*	79.5 (6.51)
Follow-up years, *M(SD)*	8.1 (4.63)
Sex, *N*(%)	
Male	321 (23.50%)
Female	1045 (76.50%)
Race and ethnicity, *N*(%)	
Hispanic	61 (4.47%)
Non-Hispanic Black	50 (3.66%)
Non-Hispanic White	1247 (91.29%)
Non-Hispanic Other	8 (0.59%)
Smoking status, *N*(%)	
Never	804 (58.86%)
Former	526 (38.50%)
Current	36 (2.64%)
ApoE4 copies, *N*(%)	
0	909 (79.32%)
1	224 (19.55%)
2	13 (1.13%)
Married, *N*(%)	510 (37.34%)
Retired, *N*(%)	1207 (88.36%)
Category of main occupation, *N*(%)	
Operative and service workers	123 (9.69%)
Managerial	675 (53.19%)
Professional	471 (37.12%)
Years of education, *M(SD)*	15.0 (3.25)
Mini-Mental State Examination Score, *M(SD)*	28.5 (1.52)

### Associations between proxy categories

**Fig. 1:**
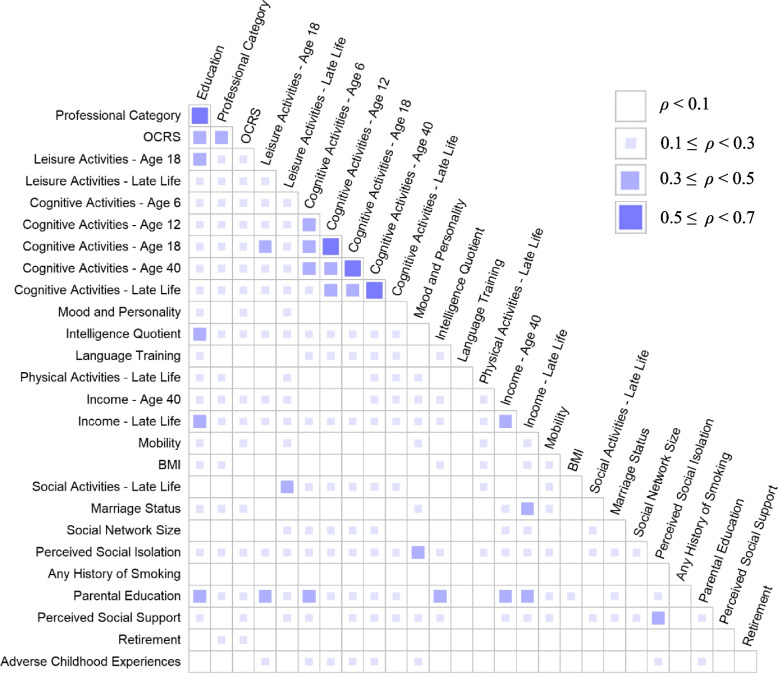
Illustration of effect sizes for correlation between operationalizations based on proxy categories

The first analysis examined the association across proxy categories, including composite scores for activity variables (Appendix 4). Few associations showed a substantial effect size (see Fig. 1). Large effect sizes were seen for four associations: education and job attainment level (*ρ* = 0.558), cognitive activities at age 12 and cognitive activities at age 18 (*ρ* = 0.642), cognitive activities at age 18 and cognitive activities at age 40 (*ρ* = 0.570), and cognitive activities at age 40 and cognitive activities in late life (*ρ* = 0.527). These findings show that greater frequency of cognitive activities at 12, 18, and 40 are associated with greater frequency of cognitive activities at 18, 40, and late life, respectively; and more years of education is associated with higher level of job attainment. Weak effect sizes (*ρ* ≥ 0.1) were seen for 165 associations and moderate effect sizes (*ρ* ≥ 0.3) were seen for 23 associations. Therefore, there were 27 associations with at least moderate effect sizes of 351 correlations (7.69%).

### Associations between observed variables

The observed variables that are classified within proxy categories also demonstrated weak associations. Proxy categories of leisure activities, social activities, and cognitive activities were composites of three to six observed variables/activities. We examined the correlation between the observed variables classified within leisure activities, within social activities, and across leisure activities and social activities, due to overlap between the activities in these categories in the literature search. All correlations among observed variables/activities for leisure and social activities had relatively minor effect sizes (Appendix 4). Of the 36 associations, 20 had a small effect size and none had moderate or large effect sizes. The strongest association among all variables was between an observed variable for leisure activities in late life and an observed variable for social activities in late life: traveling and going to restaurants/sporting events (*ρ* = 0.255).

Observed variables within the proxy categories of cognitive activities at different ages were also examined. Of the 69 associations, 10 had a moderate effect size and 1 had a large effect size (Appendix 4). The strongest association was between reading and hearing stories at age 6 (*ρ* = 0.626). Reading with going to the library and reading newspapers with reading magazines were consistently the strongest associations within age groups after age 6.

### Factor analysis

The multiphase confirmatory factor analysis systematically narrowed 54 observed variables from MAP to 22 variables which showed good fit. Details on removed observed variables are provided in Appendix 5. Due to the large number of variables and factors, each dimension was examined separately before they were combined. All dimensions showed a higher CFI, lower χ^2^, and lower SRMR in the final models, indicating the final models were improvements over the preliminary models. The final combined model showed adequate model fit: χ^2^ (158) = 434.190, CFI = 0.956, RMSEA = 0.035 (90% confidence interval: 0.031 to 0.038), SRMR = 0.030 [44, 51]. Fit indices from preliminary models to final models for each dimension and the combined model are in Appendix 5.

**Fig. 2:**
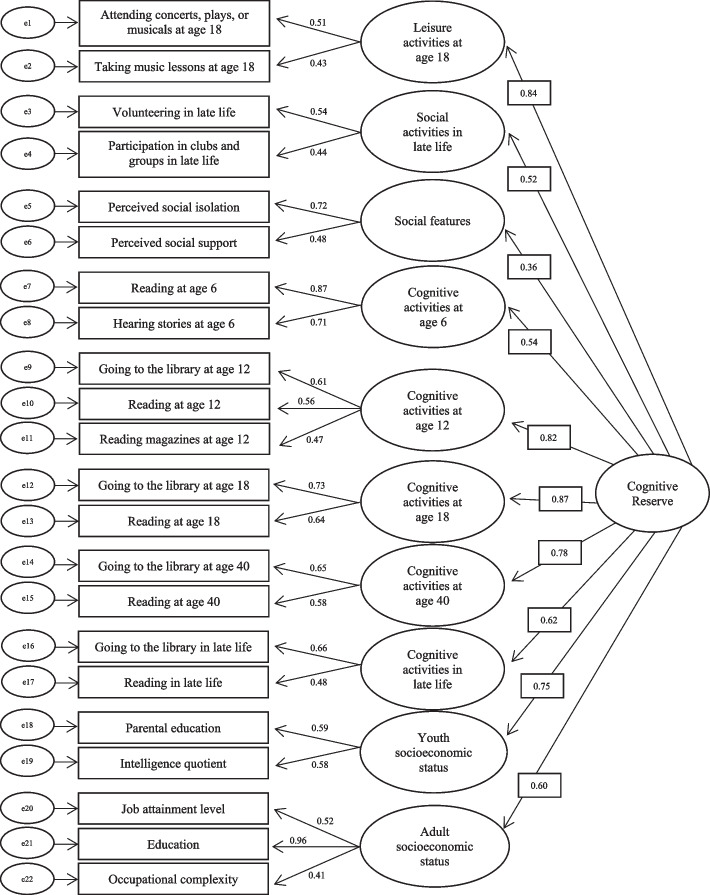
Diagram of final multiphase confirmatory factor analysis model. e1-e22 indicate the measurement error for observed variables. Values indicate the standardized loadings of variables and factors of the latent cognitive reserve factor

All variables that were retained in the final model, standardized loadings, and latent factors are shown in Fig. 2. Latent factors of the observed variables in the final model were leisure activities at age 18; social activities in late life; social features; cognitive activities at ages 6, 12, 18, 40, and in late life; youth SES; and adult SES. The standardized loadings of these factors for the overall latent CR factor ranged from 0.36 to 0.87. The largest loadings were for leisure activities at age 18 (0.84); cognitive activities at ages 12 (0.82), 18 (0.87), and 40 (0.78); and youth socioeconomic status (0.75).

Using the final model, we computed predicted values for the CR factor, creating a unique score for each participant. The score ranged from -3.20 to 2.02 (*Mdn* = 0.13, IQR [-0.44, 0.65]), based on their values on observed variables and standardized loadings of the factors of the CR construct.

### Association between operationalization and late-life global cognition

**Fig. 3:**
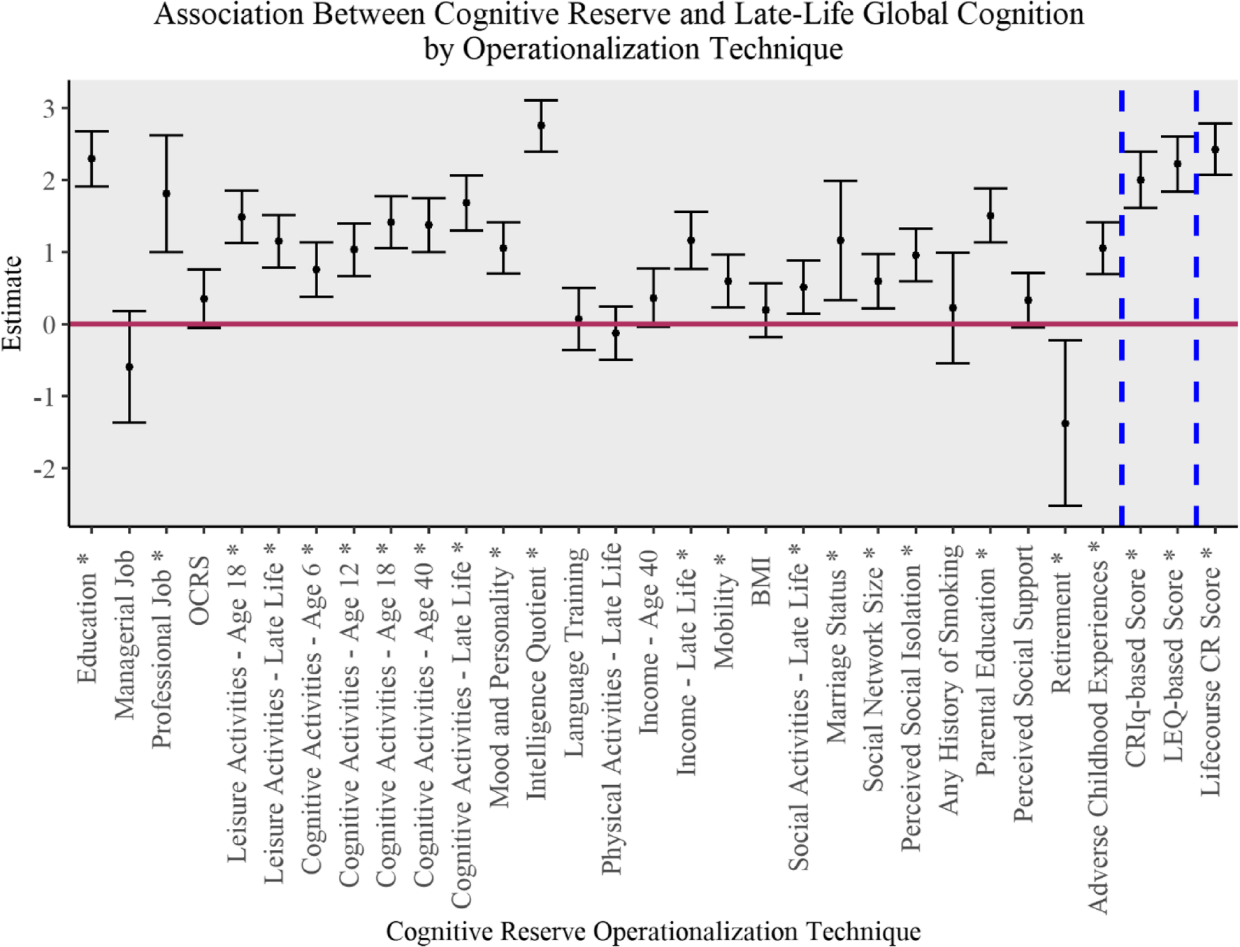
Point estimates and 95% confidence intervals for generalized linear mixed effects models for different cognitive reserve operationalization techniques and global cognition scores over the course of follow-up. Dotted lines separate proxy categories from measurement through questionnaires and the lifecourse CR score created in this study. * indicates estimates significantly different from zero. OCRS: occupational cognitive requirements score; BMI: body mass index; CRIq: Cognitive Reserve Index questionnaire; LEQ: Lifetime of Experience Questionnaire; CR: cognitive reserve

The point estimates and 95% CIs for the (standardized) CR predictors are illustrated in Fig. 3. CR predictors that showed an estimate significantly different from 0 with the Bonferroni correction are indicated by an asterisk. One standard deviation increase in these predictors was associated with higher global cognition scores over the course of follow-up, with the exception of retirement, which was associated with lower global cognition scores. There was substantial variability in the strength of association between CR operationalization and global cognition (median effect size: 0.99, *IQR*: 0.34 to 1.39). Detailed results are provided in Table 5. The highest estimates were as follows: IQ (2.75, *SE* = 0.18, *p* < 0.001), lifecourse CR score (2.44, *SE* = 0.18, *p* < 0.001), education (2.29, *SE* = 0.19, *p* < 0.001), LEQ-based score (2.22, *SE* = 0.20, *p* < 0.001), and CRIq-based score (2.00, *SE* = 0.20, *p* < 0.001).

**Table 5:** Results of generalized linear mixed effects models for cognitive reserve predictors and global cognition scores over the course of follow-up

CR Operationalization Techniques	Global Cognition		
	Estimate (95% CI)	Standard Error	*p*-value
Education	2.29 (1.91, 2.67)	0.19	< .001
Managerial level occupation	-0.60 (-1.37, 0.18)	0.40	.133
Professional level occupation	1.81 (1.00, 2.62)	0.41	< .001
Occupational cognitive requirements score	0.35 (-0.06, 0.75)	0.21	.094
Leisure activities			
Age 18	1.48 (1.12, 1.85)	0.19	< .001
Late life	1.15 (0.78, 1.51)	0.19	< .001
Cognitive activities			
Age 6	0.75 (0.37, 1.13)	0.20	< .001
Age 12	1.03 (0.66, 1.39)	0.19	< .001
Age 18	1.14 (1.05, 1.77)	0.18	< .001
Age 40	1.37 (1.00, 1.74)	0.19	< .001
Late life	1.68 (1.29, 2.06)	0.20	< .001
Mood and personality	1.05 (0.70, 1.41)	0.18	< .001
Intelligence quotient	2.75 (2.39, 3.10)	0.18	< .001
Years of language training	0.07 (-0.36, 0.50)	0.22	.751
Physical activities in late life	-0.13 (-0.50, 0.24)	0.19	.478
Income			
Age 40	0.36 (-0.04, 0.77)	0.21	.078
Late life	1.16 (0.76, 1.55)	0.20	< .001
Mobility	0.59 (0.23, 0.96)	0.19	.001
Body mass index	0.19 (-0.18, 0.56)	0.19	.321
Social activities in late life	0.51 (0.14, 0.88)	0.19	.007
Marriage status	1.16 (0.33, 1.99)	0.42	.006
Social network size	0.59 (0.21, 0.97)	0.19	.002
Perceived social isolation	0.95 (0.59, 1.32)	0.19	< .001
Any history of smoking	0.22 (-0.54, 0.99)	0.39	.565
Parental education	1.50 (1.13, 1.88)	0.19	< .001
Perceived social support	0.33 (-0.05, 0.71)	0.19	.085
Retirement	-1.38 (-2.53, -0.23)	0.59	.018
Adverse childhood experiences	1.05 (0.69, 1.41)	0.19	< .001
CRIq-based score	2.00 (1.61, 2.39)	0.20	< .001
LEQ-based score	2.22 (1.83, 2.60)	0.20	< .001
Lifecourse CR Score	2.44 (2.08, 2.80)	0.18	< .001

*CR* cognitive reserve, *CRIq* Cognitive Reserve Index questionnaire, *LEQ* Lifetime of Experience Questionnaire

### Association between operationalization and cognitive domains

**Fig. 4:**
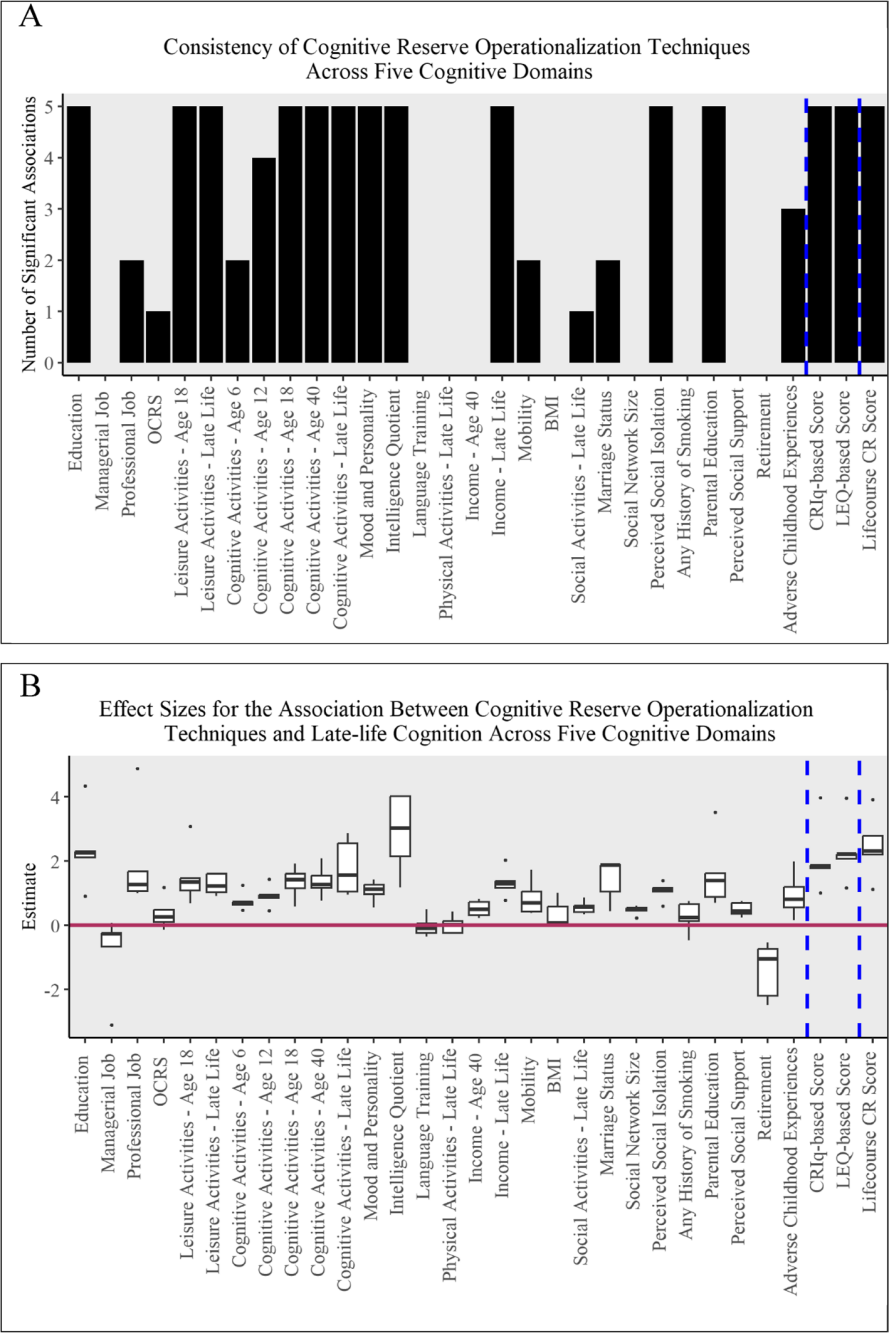
Frequency of significant associations between each CR operationalization technique and five cognitive domain outcomes (4A) and boxplots of the strength of the associations (effect sizes) between CR operationalization techniques and late-life cognition for the five cognitive domains (4B). Dotted lines separate proxy categories from measurement through questionnaires and the lifecourse CR score created in this study. OCRS: occupational cognitive requirements score; BMI: body mass index; CRIq: Cognitive Reserve Index questionnaire; LEQ: Lifetime of Experience Questionnaire; CR: cognitive reserve

In addition to global cognition, we also evaluated five cognitive domains as outcomes: episodic memory, perceptual orientation, perceptual speed, semantic memory, and working memory. The point estimates and 95% confidence intervals for the cognitive domains are provided in Appendix 6. There was not strong consistency in the association between CR operationalizations and the five cognitive domains (mean consistency: 56.1%) and variation in the effect sizes across domains (median effect size: 0.82, *IQR*: 0.40 to 1.37). The consistency of operationalization techniques for higher late-life cognition scores across the domains is provided in Fig. 4A, with effect sizes across the domains provided in Fig. 4B. As illustrated in Fig. 4B, IQ, lifecourse CR score, education, LEQ-based score, and CRIq-based score were consistently among the highest estimates. The average estimate for the 28 proxy category operationalizations was 0.91 (*SE* = 0.48), with the average estimate for IQ and education at 2.88 (*SE* = 0.49) and 2.39 *(SE* = 0.49), respectively. The average estimate for the lifecourse CR score was 2.48 (*SE* = 0.40) and the average estimate for the questionnaires was 2.21 (*SE* = 0.43). The lifecourse CR score was associated with all five domains (100% consistency) and had little variation in effect sizes (median effect size: 2.36, *IQR*: 2.21 to 2.82).

## Discussion

The study explored the operationalization of CR in the published literature and examined the effect of this variability on the association between CR and late-life cognition. Our search of the literature resulted in 20 categories of proxies that have been used in studies related to CR and which show wide variability in their degree of use and, in some cases, the experiences that are classified into these categories. We employed quantitative analyses based on the comprehensive literature review. The results revealed three key considerations for research on CR. First, among the variety of experiences that have been used as proxies of CR, there was a lack of strong association between them. Second, the association between CR and late-life cognition was highly sensitive to the technique (proxy categories, questionnaires, or a lifecourse indicator) used to operationalize CR. Third, operationalizations that include multiple experiences, including questionnaires and the lifecourse CR score developed in this study, are strongly associated with late-life cognition, demonstrating the utility of CR measures that incorporate experiences throughout the lifecourse. An awareness of qualities of operationalization and the impact of operationalization is imperative as the field of healthy aging continues to investigate the practical advantage of CR.

The results of this study revealed problems that have been suspected within the practical use of CR in research [9, 17, 56, 57]. The lack of strong associations between CR proxy categories, particularly for the chosen experiences within proxy categories, asserts that variability in the operationalization of CR stems from the experiences of choice and is exacerbated by different ways to measure those experiences. In fact, the negligible associations among proxies suggests that a change in operationalization could be measuring something different from the intended life experience. For example, within cognitive activities in late life, experiences that were not strongly associated were playing games and reading. While both were classified within the same proxy category of cognitive activities, they could more so represent personality traits or social preferences between spending time playing games with others or quiet time reading. Alternatively, it may be that the term “playing games,” is imprecise, with the potential for different meanings for different individuals. Due to the lack of strong associations, it is expected that different CR operationalization techniques were differentially associated with late-life cognition and the strength of the associations were to varying degrees. Similar differences in results was found in a study that compared different indices of CR based on education, occupation, and leisure activites [58]; and one study that compared five different operationlizations of education [17]. The results in our study extend these findings to an extensive list of CR operationalization techniques that have been utilized in the published literature. Collectively, our findings provide comprehensive support for the idea that methodological inconsistency is an important problem in the CR literature and with consequences for the field’s understanding of CR, its relationship to late-life cognition, and noteworthy CR promoting experiences.

In an effort to overcome variability in CR operationalization and weakly associated proxies, the present study utilized the CFA to develop a composite lifecourse CR score as alternative operationalization. The CFA highlighted the weak associations between different life experiences, such that over half of the observed variables in the original factors were excluded from the final model for poor fit with the other variables. Based on the observed variables that were retained through multiple iterations, several experiences, through the lifecourse, may need to be considered for CR operationalization. Similar methods have been used to create composite scores for CR in past research [23, 24]. Our study expands on these through the inclusion of more observed variables or experiences (rather than pre-created composites) than any known study. Such approaches are particularly important given that optimal life exposures for building CR or the complexity of CR regarding multiple experiences remain unclear. In fact, theorists have considered that multiple experiences could contribute uniquely to building CR and the quantity of experiences may be more important than any specific type of experience [9, 12]. These ideas are supported by the fact that the lifecourse CR score was one of the strongest and most consistent operationalization techniques associated with higher cognitive scores over the course of follow-up. Other techniques that utilize multiple experiences, the CRIq and the LEQ questionnaires, were also among the strongest and most consistent. A lifecourse approach, utilizing multiple proxies and considering the multitude of experiences that can occur across the lifespan, may be the most faithful and comprehensive representation of CR [59–61]. We urge researchers to use this approach as a basis for examining the complexity that may appropriately characterize CR in future studies.

The findings have key implications for researchers. While the CR hypothesis is a commonly used explanation for the delay in symptoms of cognitive decline, the present results reinforce concerns by theorists that the research is in a state of disorder, failing to unanimously identify the most important life experiences largely due to methodological concerns [58]. Therefore, continued research into both already-identified or new, modifiable life experiences that are theorized to enhance CR may add noise. Rather, a more effective use of resources may be a shift in focus to identifying or creating a consensus operationalization [51, 58]. Researchers must be aware of the sensitivity of results to measurement of CR, such that future studies can avoid measurement concerns [10, 58]. The current exploration of proxies offers a foundation for this future research, highlighting variables that may be particularly relevant, as well as a focus on a lifecourse approach such as a lifecourse CR score. These practical implications can aid the field in continued progress towards prevention of symptoms of cognitive decline.

The present study has several limitations. Our search of the literature, while comprehensive, does not meet criteria for a systematic review. As the goal was to examine the ways in which CR proxies have been utilized in the literature in order to inform quantitative analysis, the search can best be described as a scoping review [62]. Therefore, there is an inherent risk of bias when synthesizing research that would need to be addressed in a systematic review. The results are not intended to be used as evidence to support or refute the CR hypothesis, but for comparison based on CR operationalization and a foundation for future research. The results from our study provide a point of departure for narrowing the experiences related to CR operationalization within a lifecourse approach to CR. Furthermore, an intact model of the CR hypothesis would require an in-vivo measure of neuropathology [9, 10, 23, 63]. We did not have access to data to explore changes to results based on the inclusion of neuropathological indications. Furthermore, our approximation of CR variables utilized in the published literature is limited to variables in MAP, which does not always reflect the intended nature of a proxy. For example, bilingualism is frequently utilized as a proxy and our closest approximation was years spent learning a language. It is possible that this is not a suitable approximation of bilingualism and, thus, studies with different operationalizations may yield different results. Similarly, NART was utilized as a proxy for IQ, thereby failing to consider fluid IQ within the use of IQ in this study. While this is consistent with operationalizations of IQ in the literature, we recommend that future studies utilize crystallized and fluid IQ in order to get a more complete understanding of the potential role of IQ. Additionally, we were unable to explore novelty and nutrition, which have been identified as CR proxy categories in the literature.

Finally, there is a limit to the generalizability of these results, based on the data. First, the data is from one region in the United States, Chicago, Illinois. Therefore, the population for this study was from an urbanized, metropolitan area and the data was comprised of a high percentage of individuals identifying as Non-Hispanic White and women. Second, the population was also highly educated, with an average of 15 years of education. This further limits generalizability as this population may be more protected against symptoms of cognitive decline than the general population, depending on the degree of importance that one puts on education for building CR. Lastly, our data also had some missing observations. While we do not expect the small amount of missingness to have biased the results, it is a possibility.

## Conclusion

Inconsistent methodology in the study of CR is a major limitation of current research in this area. Our study is the most comprehensive and conclusive study to date demonstrating the challenges associated with commonly used CR proxies. We found weak associations among the variables and proxies used to operationalize CR, as well as a relationship between CR and late-life cognition that is highly dependent on the CR operationalization technique. Lifecourse measures, including questionnaires and a score that was developed in this study from multiple experiences, were among the strongest and most consistent predictors of late-life cognition. Attention to qualities of predictors, reigning in of the use of a wide variety of CR operationalizations, adherence to a lifecourse approach, and collaborative movement towards a consensus operationalization and definition of CR are imperative shifts in the study of CR that are highlighted by this study. Through the recommendation of these methodological considerations, we hope to help improve the study of risk factors related to cognitive decline and aid in the prevention of this growing public health concern.

### Supplementary Information

**Additional file 1:** **Appendix 1.** Literature search procedures. **Appendix 2.** Occupational cognitive requirements score calculation. **Appendix 3.** Names and scoring for cognitive test in global cognition score. **Appendix 4.** Correlations. **Appendix 5.** Multiphase confirmatory factor analysis model fit indices. **Appendix 6.** Cognitive domain outcomes.

## Data Availability

The data that support the findings of this study are available from Rush University Medical Center and applications requesting data can be submitted to the Rush Alzheimer’s Disease Center. Data for the current study are available from the corresponding author on reasonable request and with permission of the Rush Alzheimer’s Disease Center.
