# Supplementary material for: Systematic examination of methodological inconsistency in operationalizing cognitive reserve and its impact on identifying predictors of late-life cognition
Source: BMC Geriatr. 2023 Sep 9;23:547. doi: 10.1186/s12877-023-04263-9 (PMC10492336; doi:10.1186/s12877-023-04263-9)
Supplement: Supplementary file 1 — Additional file 1: Appendix 1. Literature search procedures. Appendix 2. Occupational cognitive requirements score calculation. Appendix 3. Names and scoring for cognitive test in global cognition score. Appendix 4. Correlations. Appendix 5. Multiphase confirmatory factor analysis model fit indices. Appendix 6. Cognitive domain outcomes. [file 12877_2023_4263_MOESM1_ESM.docx]

**Appendix 1: Literature search procedures**

A comprehensive search of the literature for CR proxies and operationalizations was undertaken and completed in January 2022. The search included articles from January 1990 to December 2021. Titles prior to January 1990 were not included to ensure consistency in the diagnosis of dementia and Alzheimer’s Disease. In the first phase, possible proxies were obtained through assessment of factors within questionnaires used to measure CR (the CRIq and LEQ), recent review articles on CR (Jones et al., 2011; Beydoun et al., 2014), and a broad literature search through three search engines. All searches were done through PsycInfo, Web of Science, and PubMed. In the search, we assessed titles and abstracts using the search “cognitive reserve” AND “dementia” or “Alzheimer’s” or “cognition” or “cognitive decline” or “cognitive impairment” or “cognitive function” or “cognitive dysfunction.” Upon identification of a new term (e.g., crossword puzzles), this term and the source of the term was noted (see Table A1). This first phase of the search uncovered 56 terms that were grouped into 20 categories. Categories were agreed upon by two researchers. A list of the terms, the source of the term, and the grouping for use in Phase II is given in Table A1.

| **Table A1.** Results of Phase I of literature search, including identified source of a term, terms, and categories for Phase II search | | |
| --- | --- | --- |
| **Source** | **Term Found** | **Search Category** |
| Halpin et al., 2021 | Adverse childhood experiences | Adverse Childhood Experiences |
| Lifetime of Experience Questionnaire | Bilingualism | Bilingualism |
| Lifetime of Experience Questionnaire | Cognitive activities | Cognitive activities |
| Sattler et al., 2012 | Reading |  |
| Murphy et al., 2014 | Crossword puzzles |  |
| Chan et al., 2019 | Playing team sports |  |
| Chan et al., 2019 | Taking vacations |  |
| Fancourt et al., 2018 | Museums |  |
| Jones et al., 2011 | Years of education | Education |
| Peeters et al., 2020 | Vocational training |  |
| Pudas & Ronnlund, 2019 | School performance |  |
| Jones et al., 2011 | Premorbid IQ | IQ |
| Boyle et al., 2021 | Verbal Intelligence |  |
| Engelman et al., 2010 | Propositional density |  |
| Farias et al., 2012 | Linguistic ability |  |
| CRIq | Leisure activities | Leisure activities |
| CRIq | Volunteer work |  |
| Lifetime of Experience Questionnaire | Music |  |
| Lifetime of Experience Questionnaire | Travel |  |
| Lifetime of Experience Questionnaire | Art |  |
| Lifetime of Experience Questionnaire | Marriage status | Marriage status |
| Conroy et al., 2010 | Boredom-proneness | Mood and personality |
| Conroy et al., 2010 | Loneliness |  |
| Geerlings et al., 2015 | Depression |  |
| Rhodes et al., 2016 | Grit |  |
| Suchy et al., 2010 | Novelty effect | Novelty |
| An et al., 2019 | Dietary | Nutrition |
| An et al., 2019 | Serum cholesterol |  |
| Beydoun et al., 2014 | Antioxidant/vitamin E |  |
| Beydoun et al., 2014 | Homocysteine |  |
| Beydoun et al., 2014 | Fatty acids |  |
| Jones et al., 2011 | Occupation level or category | Occupation |
| Carli et al., 2021 | Occupation demands or complexity |  |
| Elbejjani et al., 2017 | Parental education | Parental education |
| Beydoun et al., 2014 | Physical activities | Physical activities |
| Ihle et al., 2021 | Obesity | Physical function |
| Kujawski et al., 2021 | Functional capabilities |  |
| Eisenstein et al., 2021 | Maximal aerobic capacity |  |
| Albanese et al., 2012 | BMI |  |
| Lifetime of Experience Questionnaire | Retired | Retired |
| Conroy et al., 2010 | Social engagement | Social activities |
| Lifetime of Experience Questionnaire | Social activities |  |
| Lifetime of Experience Questionnaire | Clubs and groups |  |
| Lifetime of Experience Questionnaire | Social network | Social network |
| Glymour et al., 2008 | Social support | Social support |
| Yu et al., 2021 | Poverty | Socioeconomic status |
| Sattler et al., 2012 | SES |  |
| Meyer et al., 2018 | Neighborhood SES |  |
| Ouvrard et al., 2016 | Psychosocioeconomic precariousness |  |
| Gonzalez et al., 2013 | Childhood SES |  |
| Beydoun et al., 2014 | Coffee | Substances |
| Beydoun et al., 2014 | Caffeine |  |
| Beydoun et al., 2014 | Tea |  |
| Beydoun et al., 2014 | Alcohol |  |
| Beydoun et al., 2014 | Smoking |  |

The second phase consisted of a search for articles that used the proxies obtained in Phase I in order to determine frequency. The search described above was replicated with the addition of another phrase pertaining to the proxy of interest. For example, a search for education articles combined “cognitive reserve” and the measure of cognition with “education,” “educational,” “training,” or “school” or “schooling.” A complete list of terms utilized in the search for each proxy is provided in Table A2. Titles and abstracts were reviewed for relevance. We also made note of different operationalization among the literature for proxy categories for which we expected wide variability in the original terms retrieved in Phase I (e.g., occupation based on level or based on the demands and requirements of the work).

| **Table A2.** Search terms for Phase II of search for frequency of studies utilizing each proxy of CR from Phase I search. | |
| --- | --- |
| **Related to Cognitive Reserve and Cognitive Outcomes** | |
| cognitive reserve | |
| **AND** | |
| dementia or Alzheimer’s or cognition or cognitive decline or cognitive impairment or cognitive function or cognitive dysfunction | |
| **AND** | |
| **Search Category** | **Terms Entered** |
| Education | education or educational or training or school or schooling |
| Occupation | occupation or occupational or skilled work or complexity or supervision or work or working |
| Bilingualism | bilingualism or multilingualism or trilingualism or multilingual or bilingual or trilingual |
| IQ | premorbid IQ or verbal intelligence or propositional density or linguistic or reading test or NART or literacy or intelligence quotient |
| Leisure activities | leisure activity or leisure activities or travel or music or musical or garden or gardening or art or artist or artistic |
| Cognitive activities | cognitive activities or cognitive activity or cognitively stimulating or cognitive stimulation or puzzles or games or museums or museum or read or reading or volunteer or volunteering or team sports or team sport or vacation or vacationing or vacations |
| Physical activities | physical activity or physical activities or exercise or recreation or recreational |
| Social activities | social |
|  | **AND** |
|  | social activities or social activity or clubs or groups or social integration or social participation |
| Social network | social |
|  | **AND** |
|  | social network or social networks or social capital or friends or family or social isolation |
| Social support | social |
|  | **AND** |
|  | social support or close friends or family |
| Mood and personality | boredom proneness or boredom-proneness or boredom or loneliness or mood or personality or depression or depressive or grit |
| Socioeconomic status | socioeconomic or poverty or income or psychosocioeconomic or SES or wealth |
| Physical function | physical function or maximal aerobic capacity or obesity or functional capabilities or cardiometabolic or BMI or height or leg length or cardio or hypertension or blood pressure or grip strength or physical fitness |
| Substances | smoking or smoker or smokers or alcohol or caffeine or tea or substance |
| Diet | diet or dietary or antioxidant or vitamin or vitamins or Homocysteine or fatty acids or fatty acid or nutrition or cholesterol or nutritional |
| Parental education | parent or parental or maternal or paternal or mother or father |
|  | **AND** |
|  | education or educational or training or school or schooling |
| Adverse childhood experiences | aces or ace or adverse childhood experience or adverse childhood experiences or childhood trauma or adverse childhood |
| Marriage status | marriage status or marital or marriage or married or cohabitation or cohabitate |
| Retirement | retirement or retirement transition or retired or age of retirement |
| Novelty effect | novelty |

Excluding duplicates, 5,326 publications were retrieved in the second phase. These publications underwent review to meet final inclusion criteria. Final inclusion criteria was based on Beydoun et al., 2014 and were as follows: (1) study design as cohort or cross-sectional; (2) healthy populations or subjects with cognitive diagnoses, thereby excluding studies on particular populations, such as those with alternative ailments (3) outcome related to cognitive decline; and (4) proxy utilized as a direct proxy of CR or predictor of cognitive decline with implications for CR. Initial screening allowed for evaluation of criteria 1 and 2. Specifically, studies that were marked as review, meta-analysis, commentaries, systematic review, case-control, or case study were excluded. Additionally, as inclusion criteria required that the sample include healthy populations or those with cognitive diagnoses, rather than special groups, populations that were excluded included those with schizophrenia, psychosis, human immunodeficiency virus, acquired immunodeficiency syndrome, multiple sclerosis, Parkinson’s disease, bipolar disorder, epilepsy, stroke, brain injury, cancer, tumors, or delirium. For each proxy, articles were included if the proxy was used as a predictor of cognitive health, regardless of whether the results supported that association. A random subset of 200 articles were assessed by a second researcher for final inclusion criteria. The PRISMA flow diagram for the review is provided in Figure A1. After assessment of 1,412 retrieved articles, 753 articles met all inclusion criteria. Details on the studies described in the article, such as contextual setting, sample, and key findings, were not documented, as the purpose of the search was to identify proxies of CR, frequency of proxies, and qualities of their use (e.g., multiple operationalizations).

**Identification of studies via databases**

Records identified from:

Databases (n = 6138)

PsycInfo (n = 1368)

Web of Science (n = 1826)

PubMed (n = 2944)

Records removed *before screening*:

Duplicate records removed (n = 812)

**Identification**

Records excluded:

Ineligible due to study design other than cohort or cross-sectional or non-healthy populations (n = 3914)

Records screened

(n = 5326)

Reports sought for retrieval

(n = 1412)

Reports not retrieved:

(n = 0)

**Screening**

Reports excluded:

Not used as proxy or predictor (n = 389)

Outcome other than cognition (n = 269)

Other reason (n = 1)

Reports assessed for eligibility

(n = 1412)

Studies included in review

(n = 753)

**Included**

**Fig A1*.* Diagram of literature search in PRISMA flowchart.**

**Appendix 2: Occupational cognitive requirements score calculation**

Calculation of the occupational cognitive requirements score (OCRS) was based on the procedures from Pool et al., 2016. Using the most recent version of O*NET, we examined 10 cognitive work activities: judging qualities of things, services, people; evaluating information against standards; processing information; analyzing data or information; making decisions and solving problems; thinking creatively; updating and using job-relevant knowledge; developing objectives and strategies; scheduling work and activities; and organizing, planning, and prioritizing. For each job, the activities were associated with a score from 0 to 7 based on the level of the activity needed to perform the job. Scores were averaged across the 10 activities for an overall OCRS.

**Appendix 3: Names and scoring for cognitive test in global cognition score**

Test names for each cognitive domain and their scoring are given in Table A3.

| **Table A3.** Details of cognitive domain tests | | |
| --- | --- | --- |
| **Domain** | **Test** | **Scoring** |
| Episodic Memory | Word List Recall – Immediate | 0-30 where higher scores are indicative of more words recalled from the presentation of 10 words over three trials |
|  | Word List Recall – Delayed | 0-10 where higher scores are indicative of more words recalled from a list of 10 words after a delay of a few minutes |
|  | Word List Recognition | 0-10 where higher scores are indicative of more words recognized from 10 lists of previously-seen words mixed with novel words |
|  | East Boston Story – Immediate | 0-12 where higher scores are indicative of more parts of a story recalled |
|  | East Boston Story – Delayed | 0-12 where higher scores are indicative of more parts of a story recalled following a 3-minute delay |
|  | Logical Memory – Immediate | 0-25 where higher scores are indicative of more story units recalled |
|  | Logical Memory – Delayed | 0-25 where higher scores are indicative of more story units recalled 30 minutes after hearing a story |
| Perceptual Orientation | Line Orientation | 0-15 where higher scores are indicative of more line pairs correctly judged |
|  | Progressive Matrices | 0-16 where higher scores are indicative of correct identification of missing elements from images |
| Perceptual Speed | Symbol Digit Modalities | 0-110 where higher scores are indicative of more correctly identified symbols through the symbol’s corresponding number in a key |
|  | Number Comparison | 0-48 where higher scores are indicative of number of pairs correctly identified as the same or different minus the number incorrect |
|  | Stroop – Color Naming | 0-80 where higher scores are indicative of more colors correctly named in 30 seconds |
|  | Stroop – Word Reading | 0-80 where higher scores are indicative of more words correctly read in 30 seconds |
| Semantic Memory | Boston Naming Test | 0-15 where higher scores are indicative of more correctly named items |
|  | Category Fluency | 0-150 where higher scores are indicative of more examples generated for animals and fruits/vegetables within 60 seconds |
| Working Memory | Digit Span Forward | 0-12 where higher scores are indicative of more number sequences correctly repeated after they are read to participants, one at a time |
|  | Digits Backward | 0-12 where higher scores are indicative of more number sequences correctly repeated backwards after they are read to participants, one at a time |
|  | Digits Ordering | 0-14 where higher score are indicative of more sequences in which the participants puts the digits of the sequence in order from smallest to largest |

**Appendix 4: Correlations**

Full results from the first correlation, between proxy categories, are shown in Table A4. Results from observed variables in the categories of leisure activities at age 18, leisure activities in late life, and social activities in late life are provided in Table A5. Correlations for observed variables within the proxy categories of cognitive activities at all ages are in Table A6. Associations with moderate strength are boldface.

| **Table A4.** Correlations between all proxy categories | | | | | | | | | |
| --- | --- | --- | --- | --- | --- | --- | --- | --- | --- |
|  | **Job Attainment Level** | **OCRS** | **LA –**  **Age 18** | **LA - Late Life** | **CA - Age 6** | **CA –**  **Age 12** | **CA –**  **Age 18** | **CA –**  **Age 40** | **CA - Late Life** |
| **Education** | **.558**  **<.001** | **.420**  **<.001** | **.363**  **<.001** | .236  <.001 | .220  <.001 | .255  <.001 | .273  <.001 | .273  <.001 | .270  <.001 |
| **Job Attainment Level** |  | **.479**  **<.001** | .234  <.001 | .155  <.001 | .203  <.001 | .182  <.001 | .190  <.001 | .233  <.001 | .225  <.001 |
| **OCRS** |  |  | .120  <.001 | .142  <.001 | .104  <.001 | .163  <.001 | .106  <.001 | .133  <.001 | .133  <.001 |
| **LA - Age 18** |  |  |  | .159  <.001 | .267  <.001 | .295  <.001 | **.306**  **<.001** | .239  <.001 | .229  <.001 |
| **LA - Late Life** |  |  |  |  | .188  <.001 | .190  <.001 | .204  <.001 | .235  <.001 | .285  <.001 |
| **CA - Age 6** |  |  |  |  |  | **.459**  **<.001** | **.413**  **<.001** | **.364**  **<.001** | .280  <.001 |
| **CA - Age 12** |  |  |  |  |  |  | **.642**  **<.001** | **.470**  **<.001** | **.345**  **<.001** |
| **CA - Age 18** |  |  |  |  |  |  |  | **.570**  **<.001** | **.415**  **<.001** |
| **CA - Age 40** |  |  |  |  |  |  |  |  | **.527**  **<.001** |
|  | **Mood and Personality** | **IQ** | **Language**  **Training** | **PA –**  **Late Life** | **Income - Age 40** | **Income - Late Life** | **Mobility** | **BMI** | **SA – Late Life** |
| **Education** | .128  <.001 | **.409**  **<.001** | .135  <.001 | .157  <.001 | .280  <.001 | **.415**  **<.001** | .189  <.001 | .104  <.001 | .071  .010 |
| **Job Attainment Level** | .089  .001 | .267  <.001 | .034  .289 | .108  <.001 | .111  <.001 | .242  <.001 | .087  .001 | .130  <.001 | .083  .004 |
| **OCRS** | .110  <.001 | .133  <.001 | .041  .214 | .096  <.001 | .150  <.001 | .212  <.001 | .120  <.001 | .086  .002 | .050  .082 |
| **LA - Age 18** | .067  .012 | .241  <.001 | .075  .016 | .055  .040 | .154  <.001 | .209  <.001 | .075  .005 | .068  .013 | .092  <.001 |
| **LA - Late Life** | .158  <.001 | .129  <.001 | .013  .684 | .196  <.001 | .119  <.001 | .167  <.001 | .239  <.001 | .029  .280 | **.363**  **<.001** |
| **CA - Age 6** | .075  .007 | .122  <.001 | .128  <.001 | .085  .002 | .095  .001 | .156  <.001 | .078  .005 | .078  .006 | .167  <.001 |
| **CA - Age 12** | .045  .092 | .173  <.001 | .139  <.001 | .068  .011 | .080  .005 | .119  <.001 | .079  .003 | .031  .264 | .150  .037 |
| **CA - Age 18** | .021  .440 | .195  <.001 | .165  <.001 | .048  .077 | .070  .013 | .154  <.001 | .067  .013 | .049  .079 | .184  <.001 |
| **CA - Age 40** | .041  .125 | .199  <.001 | .189  <.001 | .117  <.001 | .113  <.001 | .198  <.001 | .093  <.001 | .079  .004 | .223  <.001 |
| **CA - Late Life** | .078  .004 | .246  <.001 | .160  <.001 | .125  <.001 | .128  <.001 | .198  <.001 | .088  .001 | .038  .171 | .266  <.001 |
| **Mood and Personality** |  | .097  <.001 | -.011  .712 | .152  <.001 | .152  <.001 | .223  <.001 | .154  <.001 | .047  .075 | .073  .008 |
| **IQ** |  |  | .190  <.001 | .030  .256 | .219  <.001 | .270  <.001 | .060  .023 | .114  <.001 | .009  .736 |
| **Language Training** |  |  |  | .045  .151 | .029  .371 | .041  .208 | .007  .820 | .075  .018 | .027  .396 |
| **PA – Late Life** |  |  |  |  | .106  .001 | .135  <.001 | .204  <.001 | .155  <.001 | .154  <.001 |
| **Income - Age 40** |  |  |  |  |  | **.395**  **<.001** | .098  <.001 | .085  .003 | .086  .003 |
| **Income - Late Life** |  |  |  |  |  |  | .198  <.001 | .139  <.001 | .092  .001 |
| **Mobility** |  |  |  |  |  |  |  | .131  <.001 | .140  <.001 |
| **BMI** |  |  |  |  |  |  |  |  | -.025  .373 |
|  | **Marriage Status** | **Social Network Size** | **Perceived Social Isolation** | **Any History of Smoking** | **Parental Education** | **Perceived Social Support** | **Retirement** | **ACEs** |  |
| **Education** | .179  <.001 | .054  .041 | .233  <.001 | -.032  .225 | **.448**  **<.001** | .174  <.001 | .059  .025 | .085  .001 |  |
| **Job Attainment Level** | .106  <.001 | .059  .029 | .160  <.001 | .030  .274 | .262  <.001 | .098  <.001 | .125  <.001 | .062  .025 |  |
| **OCRS** | .140  <.001 | .078  .005 | .150  <.001 | -.052  .059 | .212  <.001 | .126  <.001 | .139  <.001 | -.002  .933 |  |
| **LA - Age 18** | .039  .159 | .061  .023 | .159  <.001 | -.009  .750 | **.311**  **<.001** | .085  .002 | 0.02  .424 | .155  <.001 |  |
| **LA - Late Life** | .026  .335 | .160  <.001 | .184  <.001 | .040  .130 | .177  <.001 | .165  <.001 | .063  .016 | -0.01  .960 |  |
| **CA - Age 6** | .054  .056 | .114  <.001 | .173  <.001 | .043  .117 | **.310**  **<.001** | .110  <.001 | -.024  .383 | .269  <.001 |  |
| **CA - Age 12** | .058  .037 | .132  <.001 | .104  <.001 | .015  .590 | .205  <.001 | .092  <.001 | .013  .629 | .132  <.001 |  |
| **CA - Age 18** | .050  .067 | .128  <.001 | .119  <.001 | .057  .033 | .189  <.001 | .104  <.001 | -.017  .526 | .156  <.001 |  |
| **CA - Age 40** | .055  .047 | .110  <.001 | .142  <.001 | .030  .270 | .183  <.001 | .131  <.001 | -.004  .891 | .101  <.001 |  |
| **CA - Late Life** | .068  .013 | .093  <.001 | .141  <.001 | .020  .470 | .235  <.001 | .132  <.001 | -.012  .663 | .065  .020 |  |
| **Mood and Personality** | .212  <.001 | .094  <.001 | **.353**  **<.001** | .035  .183 | .085  .001 | .183  <.001 | .026  .328 | .104  <.001 |  |
| **IQ** | .091  .001 | -.008  .757 | .174  <.001 | -.061  .021 | **.309**  **<.001** | .125  <.001 | -.037  .167 | .070  .009 |  |
| **Language Training** | -.035  .279 | -.015  .619 | -.002  .942 | -.078  .012 | .081  .009 | .037  .243 | -.052  .095 | .072  .021 |  |
| **PA – Late Life** | .056  .040 | .057  .030 | .103  <.001 | -.022  .411 | .071  .007 | .058  .034 | .042  .112 | -.075  .005 |  |
| **Income - Age 40** | .206  <.001 | .143  <.001 | .216  <.001 | -.094  <.001 | **.313**  **<.001** | .193  <.001 | -.060  .031 | .031  .268 |  |
| **Income - Late Life** | **.399**  **<.001** | .111  <.001 | .218  <.001 | -.027  .317 | **.354**  **<.001** | .220  <.001 | -.006  .822 | .058  .039 |  |
| **Mobility** | .194  <.001 | .090  <.001 | .143  <.001 | .035  .176 | .113  <.001 | .154  <.001 | -.008  .752 | .012  .651 |  |
| **BMI** | .018  .526 | -.017  .526 | .003  .921 | .012  .558 | .124  <.001 | .016  .578 | -.024  .360 | .022  .415 |  |
| **SA - Late Life** | -.014  .604 | .274  <.001 | .185  <.001 | .084  .002 | .077  .005 | .145  <.001 | .007  .811 | .041  .141 |  |
| **Marriage Status** |  | .090  .001 | .145  <.001 | .011  .683 | .180  <.001 | .243  <.001 | .023  .408 | .016  .564 |  |
| **Social Network Size** |  |  | .239  <.001 | .044  .092 | .046  .082 | .226  <.001 | -.031  .234 | .096  <.001 |  |
| **Perceived Social Isolation** |  |  |  | .024  .383 | .144  <.001 | **.362**  **<.001** | .034  .217 | .123  <.001 |  |
| **Any History of Smoking** |  |  |  |  | -.090  <.001 | .001  .969 | -.054  .041 | .085  .002 |  |
| **Parental Education** |  |  |  |  |  | .146  <.001 | -.026  .321 | .127  <.001 |  |
| **Perceived Social Support** |  |  |  |  |  |  | .027  .332 | .049  .079 |  |
| **Retirement** |  |  |  |  |  |  |  | -.054  .042 |  |
| CA: cognitive activities; LA: leisure activities; SA: social activities; PA: physical activities; OCRS: occupational cognitive requirements score; BMI: body mass index; IQ: intelligence quotient; ACEs: adverse childhood experiences. | | | | | | | | | |

| **Table A5.** Observed variables for leisure activities at age 18 and in late life and social activities in late life | | | | | | | | | |
| --- | --- | --- | --- | --- | --- | --- | --- | --- | --- |
|  | **Leisure Activities – Age 18** | | | **Leisure Activities – Late Life** | | **Social Activities – Late Life** | | | |
|  | **Concerts** | **Music Lessons** | **Art Lessons** | **Volunteering** | **Traveling** | **Visiting Relatives** | **Groups and Clubs** | **Church** | **Restaurants and Sports** |
| **Concerts** |  | .219  <.001 | .184  <.001 | .124  <.001 | .150  <.001 | .058  .031 | .093  <.001 | -.022  .423 | .063  .019 |
| **Music Lessons** |  |  | .174  <.001 | .100  <.001 | .048  .076 | .002  .945 | .075  .005 | -.020  .463 | .013  .634 |
| **Art Lessons** |  |  |  | .040  .132 | .140  <.001 | .033  .221 | .116  <.001 | -.052  .059 | .034  .203 |
| **Volunteering** |  |  |  |  | .161  <.001 | .133  <.001 | .238  <.001 | .178  <.001 | .062  .019 |
| **Traveling** |  |  |  |  |  | .219  <.001 | .168  <.001 | .074  .007 | .255  <.001 |
| **Visiting Relatives** |  |  |  |  |  |  | .143  <.001 | .122  <.001 | .238  <.001 |
| **Groups and Clubs** |  |  |  |  |  |  |  | .102  <.001 | .113  <.001 |
| **Church** |  |  |  |  |  |  |  |  | .079  .004 |

| **Table A6.** Observed variable correlations for within the proxy category of cognitive activities at all ages | | | | | | |
| --- | --- | --- | --- | --- | --- | --- |
|  | **Games** | **Reading** | **Library** | **Newspaper** | **Magazine** | **Writing Letters** |
| **Stories – Age 6** | .294  <.001 | **.626**  **<.001** |  |  |  |  |
| **Games – Age 6** |  | .264  <.001 |  |  |  |  |
| **Playing Team Sports – Age 12** | .173  <.001 | -.002  .937 | .069  .010 | .132  <.001 | .131  <.001 | .026  .328 |
| **Games – Age 12** |  | .192  <.001 | .211  <.001 | .157  <.001 | .217  <.001 | .172  <.001 |
| **Reading – Age 12** |  |  | **.336**  **<.001** | .208  <.001 | .245  <.001 | .245  <.001 |
| **Library – Age 12** |  |  |  | .164  <.001 | .257  <.001 | .227  <.001 |
| **Newspaper – Age 12** |  |  |  |  | **.453**  **<.001** | .196  <.001 |
| **Magazine – Age 12** |  |  |  |  |  | **.339**  **<.001** |
| **Games – Age 18** |  | .182  <.001 | .245  <.001 | .190  <.001 | .193  <.001 | .119  <.001 |
| **Reading – Age 18** |  |  | **.449**  **<.001** | .191  <.001 | .239  <.001 | .245  <.001 |
| **Library – Age 18** |  |  |  | .149  <.001 | .202  <.001 | .243  <.001 |
| **Newspaper – Age 18** |  |  |  |  | **.495**  **<.001** | .051  .058 |
| **Magazine – Age 18** |  |  |  |  |  | .124  <.001 |
| **Games – Age 40** |  | .149  <.001 | .201  <.001 | .123  <.001 | .126  <.001 | .209  <.001 |
| **Reading – Age 40** |  |  | **.368**  **<.001** | .214  <.001 | **.311**  **<.001** | .225  <.001 |
| **Library – Age 40** |  |  |  | .175  <.001 | .210  <.001 | .282  <.001 |
| **Newspaper – Age 40** |  |  |  |  | **.338**  **<.001** | .139  <.001 |
| **Magazine – Age 40** |  |  |  |  |  | .178  <.001 |
| **Games – Late Life** |  | .048  .079 | .112  <.001 | .089  .001 | .028  .307 | .077  .005 |
| **Reading – Late Life** |  |  | **.309**  **<.001** | .091  <.001 | .102  <.001 | .168  <.001 |
| **Library – Late Life** |  |  |  | .095  .002 | .085  .002 | .164  <.001 |
| **Newspaper – Late Life** |  |  |  |  | **.336**  **<.001** | .120  <.001 |
| **Magazine – Late Life** |  |  |  |  |  | .159  <.001 |

**Appendix 5: Multiphase confirmatory factor analysis model fit indices**

The variables described here were excluded for poor fit. Volunteering in late life (λ=.228) and going to restaurants or sporting events in late life (λ=.327) were excluded from the late-life factor within the Leisure Activities dimension. These exclusions left traveling in late life as an orphan variable and it was also excluded when the models were combined. Playing teams sports at age 12 (λ=.078) was excluded from the social features factor of the Social Characteristics/Activities dimension, as well as going to church (λ=.262), going to restaurants or sporting events (λ=.321), and visiting relatives (λ=.346) from the late-life factor. Variables within each age factor were excluded from the Cognitive Activities dimension: playing games (λ=.386) from the age 6 factor; playing games (λ=.377), playing team sports (λ=.172), and reading the newspaper (λ=.347) from the age 12 factor; years of language training (λ=.101), playing games (λ=.367), writing letters (λ=.366), reading the newspaper (λ=.373), and reading magazines (λ=.397) from the age 18 factor; playing games (λ=.332), writing letters (λ=.396), reading the newspaper (λ=.333), and reading magazines (λ=.386) from the age 40 factor; and playing games (λ=.201), writing letters (λ=.371), reading the newspaper (λ=.392), and reading magazines (λ=.375) from the late-life factor. Years of language training (λ=-.105), playing team sports at age 12 (λ=.085), and ACEs (λ=.163) were excluded from youth factor of the SES dimension, along with managerial job attainment (λ=-.776) and income at age 40 (λ=.365) from the adulthood factor.

The preliminary combined model showed adequate model fit before going through additional iterations in which variables with λ <.4 were removed: taking art lessons (λ=.354) was removed from the age 18 factor of the Leisure Activities dimension; and social network size (λ=.385) was removed from the social features factor of the Social Characteristics/Activities dimension. Writing letters (λ=.456) from the age 12 factor of the Cognitive Activities dimension and income at baseline (λ=.460) from the adulthood factor of the SES dimension were also removed due to particularly weak fit with other observed variables within their respective factors.

The model fit from the preliminary model for each dimension and final model for each dimension are shown in Table A7, as well as details for the preliminary and final combined model.

| **Table A7.** Multiphase CFA model fit indices for each dimension and combined model. | | | | | | |
| --- | --- | --- | --- | --- | --- | --- |
| **Dimension** | | **DF** | **Chi-square** | **CFI** | **RMSEA** | **SRMR** |
| Leisure Activities | |  |  |  |  |  |
|  | Preliminary | 8 | 26.755 | .940 | .040 | .026 |
|  | Final | 2 | 9.520 | .959 | .051 | .019 |
| Social Characteristics/Activities | |  |  |  |  |  |
|  | Preliminary | 26 | 105.511 | .883 | .046 | .033 |
|  | Final | 4 | 19.114 | .962 | .051 | .022 |
| Cognitive Activities | |  |  |  |  |  |
|  | Preliminary | 367 | 4208.201 | .577 | .085 | .075 |
|  | Final | 40 | 227.231 | .947 | .057 | .039 |
| Socioeconomic Status | |  |  |  |  |  |
|  | Preliminary | 34 | 966.228 | .673 | .137 | .116 |
|  | Final | 7 | 63.750 | .965 | .075 | .032 |
| Combined Model | |  |  |  |  |  |
|  | Preliminary | 249 | 764.356 | .929 | .038 | .034 |
|  | Final | 158 | 434.190 | .956 | .035 | .030 |
| DF: degrees of freedom; CFI: Comparative Fit Indices; RMSEA: Root Mean Squared Error of Approximation. SRMR: Standardized Root Mean Square Residual. CFA: confirmatory factory analysis. Values of CFI ≥.95, RMSEA ≤.07, SRMR <.08 were the cutoffs established a priori as indicative of adequate model fit. | | | | | | |

**Appendix 6: Cognitive Domain Outcomes**

The point estimates and 95% confidence intervals for the cognitive reserve operationalization techniques and the five cognitive domain outcomes are provided in Table A8. Significant associations with higher cognitive scores over the course of follow-up at the level of the Bonferroni correction (*p*<.0016) are in bold.

| **Table A8.** Results for the association between cognitive reserve operationalization technique and five cognitive domains. | | | | | | | | | | | | | | | | |
| --- | --- | --- | --- | --- | --- | --- | --- | --- | --- | --- | --- | --- | --- | --- | --- | --- |
| Cognitive Reserve  Operationalization Technique | | Cognitive Domains | | | | | | | | | | | | | | |
|  |  | Episodic Memory | | | Perceptual Orientation | | | Perceptual Speed | | | Semantic Memory | | | Working Memory | | |
|  |  | Estimate  (95% CI) | SE | p-value | Estimate  (95% CI) | SE | p-value | Estimate  (95% CI) | SE | p-value | Estimate  (95% CI) | SE | p-value | Estimate  (95% CI) | SE | p-value |
| Education | | 2.26  (1.74, 2.78) | 0.26 | **<.001** | 4.34  (3.65, 5.02) | 0.35 | **<.001** | 2.12  (1.60, 2.64) | 0.26 | **<.001** | 0.91  (0.64, 1.18) | 0.14 | **<.001** | 2.30  (1.69, 2.91) | 0.31 | **<.001** |
| Managerial Job | | -0.66  (-1.69, 0.38) | 0.53 | .214 | -3.10  (-4.49, -1.71) | 0.71 | <.001 | 0.07  (-0.97, 1.10) | 0.53 | .897 | -0.26  (-0.78, 0.27) | 0.27 | .338 | -0.24  (-1.44, 0.96) | 0.61 | .692 |
| Professional Job | | 1.67  (0.58, 2.76) | 0.55 | .003 | 4.88  (3.44, 6.32) | 0.74 | **<.001** | 1.26  (0.17, 2.34) | 0.55 | .023 | 0.99  (0.44, 1.54) | 0.28 | **<.001** | 1.07  (-0.19, 2.32) | 0.64 | .096 |
| OCRS | | 0.10  (-0.45, 0.65) | 0.28 | .719 | 1.18  (0.45, 1.92) | 0.37 | **.002** | 0.48  (-0.06, 1.03) | 0.28 | .084 | 0.26  (-0.01, 0.54) | 0.14 | .063 | -0.14  (-0.77, 0.50) | 0.32 | .669 |
| Leisure Activities | |  |  |  |  |  |  |  |  |  |  |  |  |  |  |  |
|  | Age 18 | 1.46  (0.95, 1.97) | 0.26 | **<.001** | 3.08  (2.41, 3.76) | 0.34 | **<.001** | 1.08  (0.58, 1.58) | 0.25 | **<.001** | 0.68  (0.42, 0.94) | 0.13 | **<.001** | 1.35  (0.75, 1.95) | 0.31 | **<.001** |
|  | Late Life | 1.22  (0.73, 1.70) | 0.25 | **<.001** | 1.60  (0.93, 2.27) | 0.34 | **<.001** | 1.62  (1.15, 2.10) | 0.24 | **<.001** | 0.91  (0.67, 1.16) | 0.12 | **<.001** | 1.02  (0.45, 1.60) | 0.29 | **.001** |
| Cognitive Activities | |  |  |  |  |  |  |  |  |  |  |  |  |  |  |  |
|  | Age 6 | 0.71  (0.19, 1.23) | 0.26 | .007 | 1.25  (0.55, 1.96) | 0.36 | **.001** | 0.73  (0.22, 1.24) | 0.26 | .005 | 0.47  (0.20, 0.73) | 0.13 | **.001** | 0.64  (0.03, 1.25) | 0.31 | .041 |
|  | Age 12 | 0.92  (0.42, 1.42) | 0.25 | **<.001** | 1.43  (0.74, 2.11) | 0.35 | **<.001** | 0.96  (0.47, 1.45) | 0.25 | **<.001** | 0.46  (0.20, 0.71) | 0.13 | **<.001** | 0.85  (0.26, 1.44) | 0.30 | .005 |
|  | Age 18 | 1.15  (0.65, 1.65) | 0.25 | **<.001** | 1.92  (1.24, 2.59) | 0.35 | **<.001** | 1.42  (0.94, 1.91) | 0.25 | **<.001** | 0.59  (0.33, 0.84) | 0.13 | **<.001** | 1.61  (1.02, 2.19) | 0.30 | **<.001** |
|  | Age 40 | 1.17  (0.66, 1.68) | 0.26 | **<.001** | 2.09  (1.40, 2.78) | 0.35 | **<.001** | 1.26  (0.76, 1.76) | 0.26 | **<.001** | 0.77  (0.51, 1.03) | 0.13 | **<.001** | 1.55  (0.95, 2.14) | 0.31 | **<.001** |
|  | Late Life | 0.95  (0.43, 1.48) | 0.27 | **<.001** | 2.55  (1.81, 3.28) | 0.38 | **<.001** | 2.87  (2.36, 3.38) | 0.26 | **<.001** | 1.05  (0.78, 1.32) | 0.14 | **<.001** | 1.57  (0.95, 2.19) | 0.32 | **<.001** |
| Mood and Personality | | 1.13  (0.66, 1.60) | 0.24 | **<.001** | 1.42  (0.77, 2.07) | 0.33 | **<.001** | 1.27  (0.80, 1.74) | 0.24 | **<.001** | 0.55  (0.31, 0.79) | 0.12 | **<.001** | 0.97  (0.41, 1.52) | 0.28 | **.001** |
| Intelligence Quotient | | 2.15  (1.65, 2.66) | 0.26 | **<.001** | 4.03  (3.36, 4.70) | 0.34 | **<.001** | 3.02  (2.54, 3.50) | 0.25 | **<.001** | 1.18  (0.93, 1.43) | 0.13 | **<.001** | 4.01  (3.44, 4.58) | 0.29 | **<.001** |
| Language Training | | 0.06  (-0.51, 0.63) | 0.29 | .828 | -0.35  (-1.15, 0.45) | 0.41 | .390 | 0.50  (-0.06, 1.07) | 0.29 | .082 | -0.09  (-0.39, 0.21) | 0.15 | .560 | -0.24  (-0.93, 0.45) | 0.35 | .499 |
| Physical Activities in Late Life | | -0.25  (-0.75, 0.24) | 0.25 | .312 | 0.43  (-0.25, 1.11) | 0.35 | .217 | 0.13  (-0.36, 0.62) | 0.25 | .592 | 0.02  (-0.24, 0.27) | 0.13 | .902 | -0.24  (-0.82, 0.35) | 0.30 | .425 |
| Income | |  |  |  |  |  |  |  |  |  |  |  |  |  |  |  |
|  | Age 40 | 0.23  (-0.32, 0.77) | 0.28 | .414 | 0.82  (0.08, 1.55) | 0.37 | .029 | 0.73  (0.20, 1.26) | 0.27 | .007 | 0.31  (0.03, 0.59) | 0.14 | .030 | 0.50  (-0.14, 1.15) | 0.33 | .128 |
|  | Late Life | 1.16  (0.62, 1.69) | 0.27 | **<.001** | 2.03  (1.30, 2.76) | 0.37 | **<.001** | 1.38  (0.85, 1.91) | 0.27 | **<.001** | 0.79  (0.52, 1.06) | 0.14 | **<.001** | 1.30  (0.66, 1.93) | 0.32 | **<.001** |
| Mobility | | 0.43  (-0.06, 0.91) | 0.25 | .083 | 1.74  (1.08, 2.40) | 0.34 | **<.001** | 1.06  (0.58, 1.54) | 0.24 | **<.001** | 0.38  (0.13, 0.62) | 0.13 | .003 | 0.70  (0.13, 1.27) | 0.29 | .016 |
| Body Mass Index | | -0.03  (-0.52, 0.46) | 0.25 | .905 | 1.01  (0.34, 1.68) | 0.34 | .003 | 0.08  (-0.41, 0.57) | 0.25 | .746 | 0.07  (-0.18, 0.32) | 0.13 | .587 | 0.58  (0.01, 1.16) | 0.30 | .048 |
| Social Activities in Late Life | | 0.61  (0.10, 1.12) | 0.26 | .020 | 0.57  (-0.13, 1.28) | 0.36 | .109 | 0.87  (0.37, 1.37) | 0.25 | **.001** | 0.34  (0.08, 0.61) | 0.13 | .010 | 0.42  (-0.19, 1.03) | 0.31 | .174 |
| Marriage Status | | 0.44  (-0.72, 1.60) | 0.59 | .453 | 1.88  (0.30, 3.46) | 0.81 | .020 | 1.90  (0.77, 3.02) | 0.57 | **.001** | 1.06  (0.47, 1.65) | 0.13 | **<.001** | 1.88  (0.51, 3.26) | 0.70 | .007 |
| Social Network Size | | 0.62  (0.12, 1.13) | 0.26 | .016 | 0.54  (-0.15, 1.24) | 0.35 | .124 | 0.52  (0.02, 1.02) | 0.26 | .042 | 0.23  (-0.03, 0.49) | 0.13 | .081 | 0.46  (-0.13, 1.06) | 0.31 | .129 |
| Perceived Social Isolation | | 1.08  (0.57, 1.59) | 0.26 | **<.001** | 1.39 (0.70, 2.09) | 0.36 | **<.001** | 1.05  (0.55, 1.55) | 0.25 | **<.001** | 0.60  (0.34, 0.86) | 0.13 | **<.001** | 1.17  (0.56, 1.78) | 0.31 | **<.001** |
| Any History of Smoking | | 0.75  (-0.26, 1.76) | 0.51 | .144 | 0.24  (-1.15, 1.63) | 0.71 | .739 | 0.65  (-0.36, 1.65) | 0.51 | .208 | 0.13  (-0.38, 0.64) | 0.26 | .621 | -0.46  (-1.66, 0.73) | 0.61 | .445 |
| Parental Education | | 1.40  (0.89, 1.90) | 0.26 | **<.001** | 3.52  (2.85, 4.19) | 0.34 | **<.001** | 0.88  (0.38, 1.39) | 0.26 | **.001** | 0.70  (0.45, 0.96) | 0.13 | **<.001** | 1.62  (1.03, 2.22) | 0.31 | **<.001** |
| Perceived Social Support | | 0.24 (-0.28, 0.77) | 0.27 | .364 | 0.45  (-0.27, 1.16) | 0.37 | .224 | 0.75  (0.24, 1.26) | 0.26 | .004 | 0.36  (0.09, 0.62) | 0.14 | .009 | 0.70  (0.08, 1.32) | 0.32 | .027 |
| Retirement | | -0.74  (-2.26, 0.78) | 0.78 | .342 | -2.48  (-4.57, -0.39) | 1.07 | .020 | -1.04  (-2.56, 0.48) | 0.78 | .181 | -0.53 (-1.31, 0.24) | 0.40 | .178 | -2.20  (-4.00, -0.41) | 0.92 | .016 |
| Adverse Childhood Experiences | | 0.81  (0.32, 1.30) | 0.25 | **.001** | 1.99  (1.33, 2.66) | 0.34 | **<.001** | 0.56 (0.07, 1.05) | 0.25 | .024 | 0.16  (-0.08, 0.40) | 0.12 | .199 | 1.20  (0.63, 1.78) | 0.29 | **<.001** |
| CRIq-based Score | | 1.81  (1.27, 2.35) | 0.28 | **<.001** | 3.98  (3.28, 4.68) | 0.36 | **<.001** | 1.87  (1.33, 2.40) | 0.27 | **<.001** | 1.01  (0.74, 1.29) | 0.14 | **<.001** | 1.78  (1.16, 2.41) | 0.32 | **<.001** |
| LEQ-based Score | | 2.07  (1.53, 2.62) | 0.28 | **<.001** | 3.96  (3.26, 4.66) | 0.36 | **<.001** | 2.22  (1.69, 2.75) | 0.27 | **<.001** | 1.16  (0.89, 1.43) | 0.14 | **<.001** | 2.21  (1.59, 2.84) | 0.32 | **<.001** |
| Lifecourse CR Score | | 2.21  (1.71, 2.71) | 0.25 | **<.001** | 3.88  (3.21, 4.54) | 0.34 | **<.001** | 2.36  (1.87, 2.85) | 0.25 | **<.001** | 1.13  (0.88, 1.39) | 0.13 | **<.001** | 2.82  (2.23, 3.40) | 0.30 | **<.001** |
| Bold values indicate significant association with higher cognitive scores over the course of follow-up at the level of the Bonferroni correction (*p*<.0016). OCRS: Occupational Cognitive Requirements Score; CR: cognitive reserve; CRIq: Cognitive Reserve Index questionnaire; LEQ: Lifetime of Experience Questionnaire. | | | | | | | | | | | | | | | | |
